# Delayed perforation of the sigmoid colon following a major pelvic fracture

**DOI:** 10.4103/0974-2700.70770

**Published:** 2010

**Authors:** Luciano Santana-Cabrera, Fayna Rodríguez González, Manuel Sánchez Palacios

**Affiliations:** Intensive Care Unit, Universitary Hospital Insular in Gran Canaria, Las Palmas de Gran Canaria, Spain

Sir,

Unstable pelvic fractures are frequently seen after traffic accidents. Complications such as hemorrhage or viscera laceration contribute to a high morbimortality.[1] Computer tomography (CT) help us to identify their existence. We report the case of a delayed perforation of the sigmoid colon, presenting 5 days after a motor vehicle accident that was not detected in the initial CT examination.

**Figure 1:**
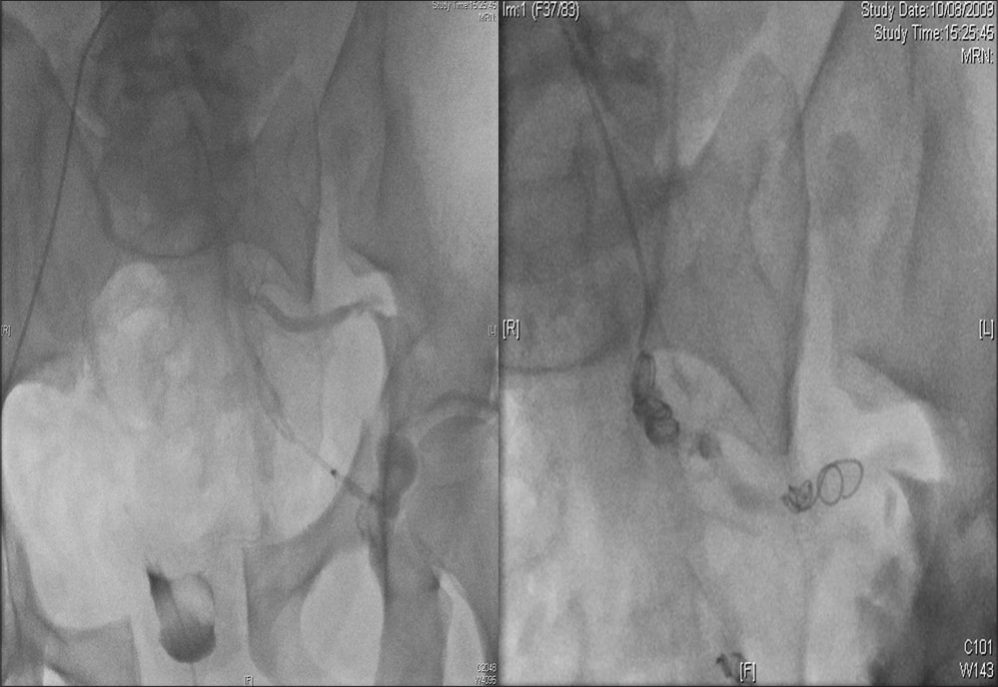
Left hypogastric artery embolization

A 53-year-old previously healthy man suffered a closed and compound pelvic fracture after a crush between two motor vehicles. On arrival to the emergency department, he was conscious and his blood pressure was 60/40 mmHg. The patient had tachycardia and tachypnea. The CT scan on admission revealed unstable compound pelvic fractures and retroperitoneal hemorrhage with signs of active bleeding, and there were no solid or hollow visceral injury. Because of active retroperitoneal hemorrhage, an angiography was made and left hypogastric artery embolization was performed [Figure 1]. On the fifth day after his trauma, the patient clinical course deteriorated developing abdominal pain, ileus, and abdominal distension associated with consciousness deterioration. His vital signs were difficult to maintain, and the patient required advanced life support and intensive resuscitation measures. After initial resuscitation, a new abdominal CT scan was made which showed pneumoperitoneum and a large amount of liquid within the peritoneal cavity. In this context, an exploration laparotomy was made which confirmed the existence of a large amount of gastrointestinal contents in the peritoneal cavity secondary to perforation of the colon at the rectosigmoid junction with necrosis at this level. The affected bowel was resected, the rectal stump was oversewn and an end colostomy was formed (Hartmann’s operation). The patient developed multiorgan failure with renal injury and anuria, needing renal replacement therapies, coagulopathy, and hemodynamic instability. After that the patient evolved favourably, so, a few days later, vasoactive drugs could be interrupted and subsequently extubation was carried out. On the – 21^st^ day, he was discharged to the ward.

Major pelvic fractures are a common sequela of traffic accidents, contributing significantly to patient’s morbimortality. Traumatic complications with colorectal injuries occurs in less than 1% of cases.[2] Early use of CT is helpful in establishing the integrity of visceras. Our case illustrates an initial computed tomography scan positive for fracture and retroperitoneal hemorrhage, but not for solid or hollow visceral injury. Explanatory causes could be the existence of a delayed traumatic perforation at the rectosigmoid junction[3] or a late perforation secondary to a intestinal infarction. Few cases of intestinal infarction after blunt trauma or rectal infarction following interventional radiology, which involves iliac arteries, have been reported.[4] Although rectum is commonly resistant to vascular insufficiency due to its multiple level blood supply, in some patients the existence of atherosclerotic disease may reduce rectum arterial supply and favor the existence of ischemia.[5] The case of our patient emphasizes that though the prevalence of occult injuries of the rectum is low and the existence of no objective physical findings can mask its appearance, careful examination should be taken in major pelvic fractures to rule out rectal tears as occurred in our patient. In conclusion, this case illustrates the importance of a careful examination in patients who has a major pelvic fracture to rule out the existence of intestinal lesions, specially in those with associated infectious syndrome. Early abdominal CT scan should be performed when there is a persisting ileus or sepsis to exclude bowel perforation.
